# Opportunistic infections in persons with HIV or other immunocompromising conditions.

**DOI:** 10.3201/eid0707.017720

**Published:** 2001

**Authors:** J E Kaplan, K Sepkowitz, H Masur, T Sirisanthana, M Russo, L Chapman

**Affiliations:** Centers for Disease Control and Prevention, Atlanta, Georgia 30333, USA. jxk2@cdc.gov


### Opportunistic Infections in Persons with HIV or Other Immunocompromising Conditions

**Jonathan E. Kaplan,* Kent Sepkowitz,† Henry Masur,‡ Thira Sirisanthana,§ Mark Russo,¶ and Louisa Chapman***

*Centers for Disease Control and Prevention, Atlanta, Georgia, USA; †Memorial Sloan-Kettering Cancer Center, New York, New York, USA; ‡Chiang Mai Medical School, Chiang Mai, Thailand; and ¶Cornell University Medical College, New York, New York, USA

Henry Masur, from the National Institutes of Health, Bethesda, Maryland, discussed the changing nature of opportunistic infections (OIs) in HIV-infected persons in the United States in light of the use of highly active antiretroviral therapy (HAART). While the incidence of nearly all OIs has decreased since 1996, several AIDS-related malignancies have maintained stable incidence rates and will likely assume greater importance. Complications resulting from infection with hepatitis C virus (HCV) will become more prevalent, since as many as 30% of HIV-infected persons are coinfected with HCV. Several "reconstitution syndromes," illnesses attributed to improved T-cell immunity, have been described as unusual manifestations of OI in the first few weeks following initiation of HAART. Antimicrobial resistance is an increasing problem for bacterial and fungal OIs and threatens to diminish the efficacy of trimethoprim-sulfamethoxazole against *Pneumocystis carinii* pneumonia, although evidence of this effect is incomplete at present. OIs also occur because many persons remain undiagnosed with HIV and therefore do not receive appropriate prophylaxis against OIs. The fact that only a minority of persons receiving HAART maintain undetectable virus levels for a sustained period suggests that HAART will not be effective in many of these patients and that OIs will increase in incidence.

Thira Sirisanthana, from the Chiang Mai Medical School, Chiang Mai, Thailand, discussed the importance of *Penicillium marneffei* (PM) infection among HIV-infected persons in Southeast Asia. Please see "*Penicillium marneffei* Infection in Patients with AIDS" on page 561 for a more detailed discussion of this topic.

Mark Russo, from Cornell University Medical College, New York, New York reviewed the consequences of HCV infection in recipients of solid organ transplants. Approximately 21,000 solid organ transplants are performed each year in the United States, and that number is increasing. In liver transplantation, persons who receive transplants to treat liver disease due to chronic hepatitis C may have lower graft survival, and retransplantation rates may be as high as 20%. Treatment of hepatitis C with interferon after liver transplantation has been disappointing, but new formulations and combination therapy with ribavirin may lead to increased efficacy. The use of hepatitis C–positive organs in hepatitis C–infected recipients is being explored. A small study of patients with chronic hepatitis C undergoing liver transplantation showed increased graft survival in those who received a hepatitis C–positive liver, compared with those who received a hepatitis C–negative liver.

Chronic hepatitis C infection is a health care problem in recipients of organs other than the liver, such as kidney transplants. Although short-term studies of less than 5 years demonstrate no difference in liver-related illness and death between kidney transplant recipients with chronic hepatitis C and those without infection, long-term studies of 10 years or more show higher illness and death rates from liver disease in HCV-infected recipients. Less is known about the effect of chronic hepatitis C in heart or lung transplant recipients, but up to 10% of recipients are hepatitis C–positive. Outcomes in recipients of these organs and the role of treatment needs to be further defined.

Louisa Chapman, from the Centers for Disease Control and Prevention in Atlanta, Georgia, spoke on xenotransplantation and xenogeneic infections. Please see "Xenotransplantation: Benefits and Risks" on page 545 for a more detailed article on this portion of the panel.

Address for correspondence: Jonathan E. Kaplan, Centers for Disease Control and Prevention, 1600 Clifton Road, Mailstop D21, Atlanta, GA 30033, USA; fax: 404-639-0910; e-mail: jxk2@cdc.gov

